# Diversity and Safety of Acupotomy Treatments for Lumbar Spine Disorders in South Korea: A Review of Clinical Studies

**DOI:** 10.3390/healthcare13101141

**Published:** 2025-05-14

**Authors:** Yubin Bae, Euijin Son, Sooyoon Lee, Younbyoung Chae, Sang-Hoon Yoon, Jungtae Leem, Seunghoon Lee, In-Seon Lee

**Affiliations:** 1 College of Korean Medicine, Kyung Hee University, Seoul 02447, Republic of Korea; becky0328@naver.com (Y.B.); novelbook@khu.ac.kr (E.S.); didalux@khu.ac.kr (S.L.); ybchae@khu.ac.kr (Y.C.); 2 Chung-Yeon Korean Medicine Clinic, Gangnam-gu, Seoul 61949, Republic of Korea; chin9yaaaa@gmail.com; 3 Research Center of Traditional Korean Medicine, College of Korean Medicine, Wonkwang University, 460, Iksan-daero, Sin-dong, Iksan 54538, Republic of Korea; julcho@naver.com; 4 Department of Acupuncture & Moxibustion, College of Korean Medicine, Kyung Hee University, Seoul 02447, Republic of Korea

**Keywords:** acupotomy, miniscalpel acupuncture, lumbar spine disorders, safety reporting guidelines, clinical practice guidelines

## Abstract

**Background**: Acupotomy is a modern acupuncture technique using a knife-shaped needle for pain treatment, combining traditional and anatomical knowledge. This study evaluates the diversity and safety of acupotomy procedures for lumbar spine disorders to aid in developing safety and reporting guidelines for clinical studies. **Methods**: A literature search was conducted on 30 October 2023 in PubMed, five Korean databases, and relevant journals with keywords like ‘low back pain’ and ‘acupotomy’. The search included clinical trial articles in English or Korean on lumbar spinal disorders treated by acupotomy. We reviewed 22 clinical studies involving 731 Korean patients published from 2008 to 2023. Data extracted included disease types, diagnosis methods, treatment specifics, needling factors, anesthesia, clinical outcomes, and safety reports. **Results**: Most studies focused on lumbar herniated discs and stenosis, with acupotomy performed on damaged tissue sites. Treatment frequency varied, and outcomes included pain scales and imaging assessments. Reporting gaps were found in needle size, insertion depth, and anesthesia status. Safety measures were poorly documented, with only six studies addressing safety and two reporting adverse events. **Conclusions**: There is a critical need for standardized clinical and reporting guidelines for acupotomy, akin to acupuncture’s existing guidelines, to enhance research consistency and quality. Future studies should develop guidelines covering target tissues, needle details, techniques, anesthesia, and adverse effects to improve acupotomy safety and effectiveness.

## 1. Introduction

**Figure 1:**
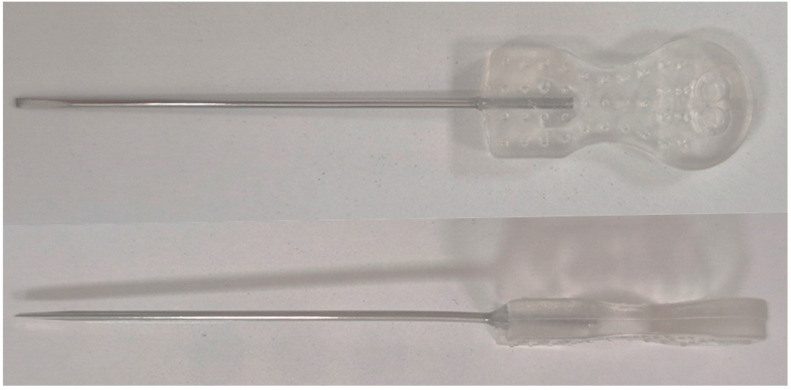
Front and side view of acupotomy needle (Dongbang medical, Seongnam, Republic of Korea; needle size 1.0 × 50). The acupotomy needle features a sharp, knife-like tip at the end, which gives it a unique shape compared to a standard acupuncture needle.

Acupotomy is an innovative acupuncture technique that integrates traditional acupuncture practices with modern anatomical knowledge, thus not necessarily adhering to traditional medical principles such as the concept of acupoints and meridians. This therapy involves a needle with a knife-shaped tip that is wider and thicker than a fine acupuncture needle to dissect, cut, or detach adhesions in soft tissues (Figure 1), and primarily treats chronic pain disorders resulting from tissue damage [1]. This technique stems from a long, thick needle resembling a spear, one of the ‘nine classical needles’ described in the ‘Huangdi Neijing’. Specifically, it is designed for lancing abscesses, treating carbuncles and furuncles, and performing bloodletting. In 1976, Hanzhang Zhu modernized this procedure using an acupotomy needle consisting of a handle, needle body, and blade and transformed it into the technique used today [2]. The procedure is relatively simpler than typical surgical procedures, causes minimal tissue damage, and carries a lower risk of infection compared to open surgery. The technique is straightforward, quick, and less painful than traditional surgery, providing strong stimulation to muscles, tendons, and ligaments, and easily separating adhered soft tissues. Acupotomy has recently been employed in China and the Republic of Korea to address musculoskeletal disorders, especially those resulting from cumulative damage like disc herniation [3], neck pain [4], and knee osteoarthritis [5]. Minimally invasive techniques that share clinical indications with acupotomy include Intra-Muscular Stimulation (IMS), which relieves chronic pain by stimulating deep muscle layers [6], and prolotherapy, which promotes soft tissue regeneration by injecting proliferative solutions into damaged ligaments and tendons [7]. Notably, this technique has been predominantly applied to the lumbar and cervical regions.

Acupotomy delivers a more intense level of stimulation than conventional acupuncture and demonstrates its efficacy in the dissection of soft tissue adhesions [8]. Compared to a surgical scalpel, it is less invasive and presents a lower risk of complications, making it a clinically valuable therapeutic modality. One of its key advantages is its ability to achieve significant therapeutic effects through minimally invasive access to pain-generating structures. While acupotomy is traditionally based on the framework of meridians and acupoints, in clinical practice, it is commonly applied to areas identified through the practitioner’s palpatory diagnosis. These areas often correspond to Ashi points or extra-meridian points, targeting muscles, ligaments, and other soft tissue structures associated with pain. Acupotomy is usually applied to these anatomical targets either to release adhesions or to stimulate dysfunctional tissues. The procedure is generally administered repeatedly over several sessions, rather than as a single treatment, depending on the patient’s condition and disease characteristics. Prior to treatment, strict sterilization and adherence to clean needling technique are essential. Post-treatment care includes compression and antiseptic management of the puncture site to prevent bleeding, hematoma, and infection. Local adverse effects such as bruising, pain, and paresthesia may occur, and in rare cases, systemic symptoms like dizziness or fatigue may also be observed.

However, like other invasive medical procedures, the acupotomy treatment process should adhere to strict guidelines to prevent adverse effects, minimize unnecessary tissue damage, reduce painful sensations in patients, and lower the risk of infection. Moreover, the specifications of the needle used (e.g., length, diameter, and width and thickness of the blade part) along with the stimulation parameters (e.g., the depth of needle insertion, the number of stimulation sites, and the number of needle manipulations including insertions and removals) significantly affect the intensity of the stimulation. The dose–response relationship is one of the most important yet challenging issues that remains unresolved in acupuncture research [9], and it is also a concern in the context of acupotomy. For example, for acupuncture treatment, various safety guidelines have been distributed and Standards for Reporting Interventions in Clinical Trials of Acupuncture (STRICTA) guidelines were designed to improve the completeness of reporting in clinical trials involving acupuncture [10]. As the acupotomy procedure can be more invasive than fine needle acupuncture, it requires its own safety and reporting guidelines. The lack of specific guidelines for reporting the diversity and quality of acupotomy procedures, as well as adverse events, creates a gap in research. Further research is needed to understand the diversity of acupotomy procedure reporting and to effectively report and investigate the safety of these procedures and their potential adverse effects.

This study aims to (1) assess the diversity of and safety reporting in acupotomy therapy procedures for lumbar spine disorders in South Korea, and to (2) compile essential information that will support the development of safety and reporting guidelines for clinical studies involving acupotomy treatments in the near future.

## 2. Methods

To analyze clinical trials including acupotomy treatment for lumbar spine disorders in South Korea, this study reviewed articles and organized and discussed the publication year, study participants, acupotomy treatment details, and clinical outcome assessment tools. We conducted a systematic search strategy following the Preferred Reporting Items for Systematic Reviews and Meta-Analyses (PRISMA) to identify relevant studies. The literature search, conducted on 30 October 2023, utilized academic research information services and PubMed with the search string: ‘(low back pain [Title/Abstract] OR lower back pain [Title/Abstract] OR lumbar pain [Title/Abstract] OR lumbago [Title/Abstract]) AND (acupotomy [Title/Abstract] OR acupotomology [Title/Abstract] OR “needle knife” [Title/Abstract] OR knife [Title/Abstract] OR scalpel [Title/Abstract] OR miniscalpel [Title/Abstract] OR “stiletto needle” [Title/Abstract] OR “sword-like needle” [Title/Abstract])’. Additionally, articles were searched using related keywords in the Journal of Korean Medical Society of Acupotomology and Korean Journal of Traditional Knowledge as well as in five Korean databases [KoreaMed, Research Information Service System (RISS), Korean Studies Information Service System (KISS), Database Periodical Information Academic (DBpia), and Oriental Medicine Advanced Searching Integrated System (OASIS)]. We also conducted a hand search of the citations in the selected papers. From those identified through electronic and manual searches, articles were excluded if they were duplicates, unrelated to lumbar spinal disorders, were not original clinical studies (e.g., reviews, meta-analyses, or protocols), or were not written in English or Korean. Two reviewers (YB and ES) independently screened articles based on titles, abstracts, and full texts for eligibility according to the inclusion criteria. Data were independently extracted from selected articles by the two reviewers and cross-checked for accuracy. Disagreements were resolved through discussions between the two reviewers and, if necessary, with a third reviewer (ISL), a doctor of Korean Medicine specializing in meridians and acupoints.

## 3. Results

### 3.1. Results of the Search

**Figure 2:**
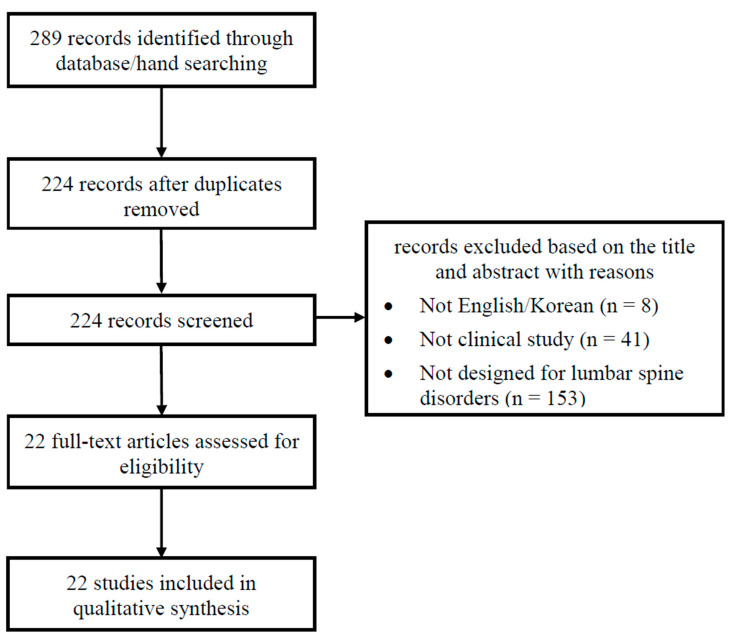
Flow diagram of literature search.

A flowchart based on the PRISMA guidelines is shown in Figure 2. The search retrieved 289 records, and 224 records were identified after removing duplicates. When reviewing the titles and abstracts, all but 22 articles were excluded from further full-text assessment due to various reasons: not written in English or Korean (n = 8); not original clinical studies (n = 41); unrelated to lumbar spine disorders (n = 153). Ultimately, we included 22 studies in the review.

### 3.2. Participants in Included Clinical Trials

The included studies, published between 2008 and 2023, comprised 22 clinical studies: 2 randomized controlled trials, 2 controlled clinical trials, 1 clinical trial, 7 case series, and 10 case reports, involving a total of 731 patients with lumbar spine disorders. The majority of the studies, seventeen in total, involved fewer than 10 patients each. Fifteen studies included patients with lumbar herniated intervertebral discs (HIVD) [11,12,13,14,15,16,17,18,19,20,21,22,23,24,25]. Two studies included patients with lumbar stenosis [26,27], and one study each included patients with fractures and sprains [28], ankylosing spondylitis [29], acute myelitis [30], and abnormal spinal curvature [31]. Additionally, one case series encompassed various conditions [32] (Table 1).

Four papers included control groups: Yun et al. [14] compared the effects of combining acupotomy with acupuncture to those in an acupuncture-only group, Park et al. [13] assessed the effects of combining acupotomy with spin decompression against those in an acupotomy-only group, and Wang et al. [16] examined the effects of combining acupotomy with herbal medicine in contrast to an herbal medicine-only group. Sung et al. [22] evaluated the effects of acupotomy compared to an acupuncture group.

### 3.3. Reporting of Treatment Procedures

#### 3.3.1. Diagnosis

Out of 22 studies, 21 used imaging techniques such as magnetic resonance imaging (MRI), computed tomography (CT), and X-ray to diagnose lumbar spine diseases. MRI was also employed to evaluate treatment responses [11,19,20,25], for example, by measuring the volume of extruded discs.

#### 3.3.2. Acupotomy Treatment Procedures

In the majority of studies (21 out of 22), detailed descriptions of the acupotomy treatment procedures and infection prevention measures were provided. In most cases, the patient’s position, disinfection of treatment sites, methods of penetration, depth of needle insertion, and methods of stimulation were described (Supplementary Table S1). Acupotomy was primarily performed on damaged tissue sites, with the frequency and number of treatments varying from 1 to 127 times. In 10 studies [13,14,15,16,17,18,24,27,30,31], acupotomy needles penetrated acupoints, and in eight studies [12,15,18,23,24,25,28,31], they targeted Ashi or tender points.

In 17 out of 22 studies (77.27%), the number of acupotomy needles inserted at each treatment session was not reported [11,15,16,17,19,20,22,23,24,25,26,27,28,30,31,32]. Regarding the size of the acupotomy needle, only five studies (22.73%) [13,14,21,24,26] provided complete details on all three critical dimensions: length, diameter, and width of the needle’s knife portion. The depth of needle insertion was reported in six studies (27.27%), ranging from 0.5 to 3 cm [14,18,20,27,28,29]. Only six studies (27.27%) provided details on anesthesia. One study did not administer anesthesia to the patients [12], three studies used lidocaine [18,26,29], and two studies applied a cream containing lidocaine and prilocaine [22,27].

### 3.4. Reporting Clinical Outcomes, Safety, and Adverse Events

The most commonly reported clinical outcomes included the pain numerical rating scale (NRS) in 14 papers, Oswestry Disability Index (ODI) in 13 papers, range of movement (ROM) in 10 papers, visual analogue scale (VAS) in 6 papers, and MRI in 4 papers. Notably, more recent studies have increasingly utilized diagnostic imaging tools such as MRI to monitor treatment effects (e.g., extruded disc volume). In most studies, it was confirmed that the intensity of pain or ROM improved after acupuncture treatment. In studies that used MRI to confirm improvement in diseases or symptoms due to acupuncture, it was often simply stated as ‘improvement was also confirmed radiologically on L-spine MRI examination’ [11] or ‘the improvement in radiology’ [19] without detailing which specific areas showed improvement or how they improved radiologically. Alternatively, it was stated as ‘the volume of extruded disc in MRI images was reduced’ [20] or ‘substantial resorption of the respective herniated disc’ [25].

Safety measures were reported in only six of the 22 papers [13,16,18,22,23,27], and these frequently lacked specific and clear indicators to confirm safety. Safety-related measures reported included methods for preventing infection, monitoring pain and other sensations, observing erythema and bleeding, and conducting blood tests. One study used a surgical safety checklist and conducted blood tests when adverse events occurred [18], suggesting that a specified safety checklist for acupotomy treatment should be developed in the near future. Out of the 22 papers reviewed, only two papers reported adverse events [12,20]. For example, Jang et al. [12] reported that the patient experienced a moderate level of discomfort and a slight increase in pain, which were relieved in a day. Park et al. [20] reported that the patient suffered from pain at the treatment sites, but without any restrictions in daily life. Four papers noted no adverse events [18,22,23,27] (Supplementary Table S2).

## 4. Discussion

We reviewed 22 clinical studies involving 731 Korean patients with various lumbar spine disorders. We found that MRI has increasingly been used in recent years to assess treatment efficacy by measuring changes in affected regions comparing pre- and post-treatment MRI scans. Acupotomy primarily targeted damaged tissue in the lumbar area but also focused on acupoints. While a significant portion of studies reported the number of needles (77.27%), over 70% did not report details on needle size (22.73%), needle insertion depth, or anesthesia status (27.27%). Furthermore, despite the critical importance of needle manipulation intensity (such as the number of lifting–thrusting techniques and the depth of needle insertion) for the dose–response relationship, it was rarely reported in clinical studies. Clinical outcomes focused on pain and function of the lumbar spine, yet the reporting of safety measures and adverse events was inconsistent. For example, acupotomy treatment may cause discomfort, pain, bleeding and bruising, infection, tissue damage, scar formation, and nerve damage (e.g., spinal cord injury); however, only discomfort and pain symptoms were reported in detail. In addition, where adverse effects were reported, only the presence and type of adverse effects were documented, with limited reporting on severity, course and prognosis, and other detailed information regarding the adverse effects. This underscores the need for standardized reporting and safety protocols for acupotomy treatment, as well as clinical practice guidelines, in the near future.

After reviewing current clinical studies on acupotomy treatments for lumbar spine disorders, it is clear that specific guidelines for reporting and safety are essential for both clinicians and researchers. While several studies had detailed acupotomy procedures even including MRI images and illustrations, a comprehensive set of reporting guidelines, similar to those used for acupuncture such as STRICTA, would greatly improve our understanding of the intervention’s rationale, the practitioner’s qualifications, co-interventions, the treatment environment, and control interventions. It might be beneficial to tailor the STRICTA guidelines to better reflect the distinct aspects of acupotomy. For instance, since acupotomy needle features a knife-shaped tip, it is crucial to report not only the length and diameter of the needle but also the width and thickness of the knife part. Additionally, details on anesthesia methods and thorough documentation of any adverse events are critical components that should be included in the checklist for acupotomy procedures. For instance, while mild bleeding and discomfort at treatment sites might not be classified as serious adverse events, the presence of erythema and swelling along with these symptoms could indicate potential infection or inflammation. Therefore, documenting adverse events, post-treatment measures, and follow-up actions is essential, regardless of whether the adverse events are mild or severe. In addition to developing acupotomy-specific safety and reporting standards, referring to established frameworks from other minimally invasive therapies may offer practical insights. For example, prolotherapy—used to promote soft tissue regeneration by injecting irritant solutions into damaged ligaments or tendons—has accumulated safety protocols and standardized reporting procedures through clinical trials and systematic reviews [33].

In clinical settings, one of the major challenges is the absence of objective criteria for determining appropriate dosage intensity during acupotomy procedures. Clinicians currently rely on subjective measures—such as patient-reported tenderness and procedural pain—to guide decisions regarding the depth of needle insertion, intensity of stimulation, and the number of targeted sites. However, these parameters remain largely unstandardized and difficult to quantify. To address this gap, future studies should incorporate digital technologies such as pressure sensors, real-time ultrasound, and patient feedback systems to quantify stimulation intensity and monitor procedural consistency in real time. Moreover, given acupotomy’s relatively stronger stimulation and more invasive nature compared to conventional acupuncture, it is essential to identify patient subgroups who are most likely to benefit from this modality. Factors such as the chronicity of pain, structural abnormalities confirmed through imaging, or the clarity of tissue pathology may help determine clinical appropriateness. Utilizing diagnostic tools such as X-ray or MRI to stratify indications and develop data-driven, personalized application criteria will be critical for optimizing both therapeutic efficacy and safety in acupotomy treatment. In parallel, future research should also focus on developing multi-dimensional post-treatment evaluation indices that go beyond subjective symptom improvement to include quantifiable changes in pressure/pain thresholds and structural improvements confirmed by imaging.

We found that acupotomy treatments predominantly target adhesions within the low back, along with the adjacent muscles and ligaments. Notably, 16 of the 22 studies also targeted acupoints, tender points, and Ashi points. This indicates that while acupotomy treatments rely heavily on anatomical knowledge and are generally aimed at damaged tissues, clinicians are also applying techniques to stimulate specific acupoints. Specifically, treatments often involved acupoints along the Bladder and Governor Vessel meridians in the lumbar region, such as BL21-26 and GV3. As the technique of selecting local acupoints (acupoints located near the affected areas) is widely used to manage pain, it may enhance the pain-relieving effects of acupotomy on damaged tissues. In future studies, the exploration of optimal treatment sites, such as comparing the effects of acupotomy stimulating both affected regions and nearby acupoints with the effects of acupotomy targeting adhered tissues alone (while controlling for the number of needles and dose of stimulation), holds promise for enhancing treatment outcomes and minimizing adverse events in patients with lumbar spine disorders.

Most studies involved co-interventions in conjunction with acupotomy, which could have made it challenging to evaluate the effectiveness, safety, and adverse events of acupotomy alone. In addition, acupotomy treatment procedures varied among studies, and long-term follow-up investigations were rare, which indicates that additional research and consideration are needed in this area. More recent papers have increasingly used imaging tools such as MRI to evaluate the effects of treatment, suggesting that future acupotomy procedures might also be assessed using such diagnostic tools, suggesting that more precise and agreed-upon formats could evolve. For example, Wang et al. applied ultrasound to guide needle insertion while performing acupotomy procedures [16], and Yoon et al. measured the safe depth for acupotomy treatment in the lumbar spine using MRI [34]. From our perspective, MRI-guided safe treatment protocols and ultrasound-guided acupotomy procedures will become more common in clinics in the near future.

In terms of safety indicators, only 6 out of 22 papers (27.27%) addressed relevant content. Acupotomy, being a tool with significant invasiveness capable of detaching adhered tissues in the body, highlights the necessity for researchers to establish a consensus on safety indicators or checklists to confirm the safety of the procedure. The findings from this and a previous study [4] indicate that the data on adverse events related to acupotomy treatment are generally insufficient. We recommend the development of tools for assessing the safety of acupotomy, including establishing clear criteria for evaluating its safety and reporting adverse effects. These should focus on understanding the physiological changes acupotomy induces in the human body. Providing guidelines for future safety research papers on acupotomy presents challenges; therefore, discussions should involve experts in the field to identify necessary elements for such guidelines.

This study has several limitations. It only included clinical studies applying acupotomy treatments to lumbar spine disorders, which limits the generalizability of our findings. Reviewing the current state of clinical research on acupotomy applied to other diseases, such as knee arthritis, could provide broader insights. Based on our findings, we plan to conduct comprehensive research to fill the gaps identified in reporting needle size, needle insertion depth, and anesthesia status in acupotomy therapy. Additionally, we will develop and propose standardized safety and reporting guidelines to improve the consistency and quality of future clinical studies involving acupotomy treatments.

## 5. Conclusions

Our review of 22 clinical studies with 731 Korean patients with lumbar spine disorders highlights the increased use of MRI for treatment assessment and a preference for acupotomy targeting both acupoints and damaged tissue. However, inconsistencies in reporting crucial details and safety highlight the need for standardized clinical and reporting guidelines for acupotomy. Future research should focus on creating practical guidelines for safe, effective acupotomy procedures, detailing target tissues, needle specifications, and protocols for adverse effect reporting. Establishing objective criteria for stimulation dosage and incorporating digital technologies, such as pressure sensors and ultrasound guidance, could enhance procedure accuracy and reproducibility. Stratifying patients by imaging-confirmed abnormalities and symptom chronicity could further optimize treatment, while developing outcome indices that combine symptom relief and objective measures will be essential for comprehensively evaluating acupotomy efficacy.

## Figures and Tables

**Table 1:** Characteristics of clinical studies using acupotomy for lumbar spine disorders in South Korea.

No. Author (Year)/Type	Diagnosis/Diagnosis	Acupotomy (No., Sex, Age)	Treatment Frequency (Total Session)	Tissue Damage	Sites/No. of Needle/Size of Needle (Length, Diameter, Thickness) (mm)	Anesthesia	Outcomes
		Control (Intervention, No., Sex, Age)					
1. Lee et al. (2008)/case series [21]	HIVD/ MRI	3 (M: 1), mean 53	1–2/10 d (1–2)	Ligament, proliferated/hardened tissues, tender points of spine, process, muscle fascia, herniated/extruded discs	Sites of tissue damage/5–15/70 × 1.0 × 0.8	NR	VAS, site of pain, gait performance
		-					
2. Kwak and Hong (2008)/case series [24]	HIVD/X-ray, MRI	4 (F: 4), mean 58.25	(1)	Narrowed intervertebral space	Ligamentum flavum, tender points/NR/70 × 1.0 × 0.8	NR	VAS, ODI, satisfaction
		-					
3. Jang et al. (2008)/case report [12]	HIVD/MRI	1 (F), 48	2/w (3)	Degenerative discs	Ashi and tender points at BL23, BL24, BL25, GV3, EX-B2, etc./2–3/NR × NR × 0.8	Not used	Neurological examination (SLRT, Milgram test, etc.), pain, ODI
		-					
4. Yun et al. (2010)/RCT [14]	HIVD/CT, MRI	Acupotomy + ACU; 33 (M: 20), 51.21 ± 13.97	1/w (1–2)	Proliferated/hardened tissues, tender points, muscle fascia, herniated/extruded discs	Sites of tissue damage/5–15/70 × 1.0 × 0.8	NR	VAS, ODI, Odom’s degree
		ACU;30 (M: 16), 50.23 ± 12.34					
5. Jung et al. (2012)/case series [26]	stenosis/MRI, fluoroscopy (X-ray)	3 (M: 2), mean 59.33	(1)	Protruded discs, spinal stenosis	Transverse process, facet joint, lamina of vertebral arch, ligamentum flavum/NR/100 × 1.0 × 0.8 (I-4 type), 80 × NR × 0.5, 80 × NR × 1.0	Lidocaine	NRS, ODI, Odom’s criteria
		-					
6. Park et al. (2012)/CCT [13]	HIVD/MRI	Acupotomy + spine decompression; 20 (M: 10), 43.20 ± 11.78	(1–3)	Proliferated/hardened tissues, herniated/extruded discs, sacroiliac joint, greater sciatic notch, ischial spine, muscle fascia	Sites of tissue damage/5–15/75 × 0.5 × 0.4	NR	VAS, ODI
		Acupotomy; 20 (M: 11), 46.10 ± 11.06					
7. Sung et al. (2013)/CCT [22]	HIVD/CT, MRI	Acupotomy + Gwanchim; 80 (M: 40), mean 47.37	(1)	NR	EX-B2, joint capsule of facet joint/NR/75 × 1.0 × NR	Lidocaine + prilocaine	NRS
		ACU;23 (M: 10), mean 40.82					
8. Yuk et al. (2013)/clinical trial [27]	stenosis/MRI, X-ray	437 (M: 172), 65 ± 10	1/d (1–3)	NR	Facet joint, erector spinae, intertransverse ligament, ligamentum flavum/NR/75 × 1.0 × NR	Lidocaine + prilocaine	NRS, ODI, global assessment
		-					
9. Kim et al. (2014)/case series [32]	Various */NA/CT, MRI	5 (M: 4), mean 55.8	1/3–7 d (1–2)	Bulging/herniated discs, annular tears, spondylolisthesis, spinal stenosis	Transverse process, BL24, BL25, BL26/NR/75 × 1.2 × NR	NR	NRS, ODI, NDI, ROM
		-					
10. Kim et al. (2015a)/case series [23]	HIVD/CT, MRI	7 (M: 5), mean 44.4	1/3–5d (1–3)	Herniated discs, spinal stenosis	Adherent sites, tender points/NR/75 × 1.2 × NR	NR	NRS, ODI, ROM
		-					
11. Kim et al. (2015b)/case series [18]	HIVD/CT, MRI	5 (NR), mean 47.2	3/2w (3)	Extruded/protruded/degenerative diffuse/bulging discs, annular tear	20–30 mm away from the spinous process, tender points in inner core muscles/3/75 × 1.2 × NR	Lidocaine	NRS, ROM, SLRT, ODI, SF-36
		-					
12. Choi et al. (2017)/case series [28]	Fracture and sprain/CT, X-ray	3 (M: 1), 56.67	1/2d (1–2)	Fracture, sprain	Adherent sites, tender points on the erector spinae/NR/50 × NR × 0.5	NR	NRS, ROM, RMDQ, EQ-5D, satisfaction with the treatment
		-					
13. Park et al. (2018)/case report [20]	HIVD/MRI	1 (F), 46	1, 2, 5/w (127)	Bulging/diffuse/extruded disk, annular tear, compression of thecal sac	Acupoints on GV meridian, facet joints/NR/50 × NR × 0.5	NR	NRS, MRI
		-					
14. Lee et al. (2019)/case report [25]	HIVD/MRI	1 (M), 48	2–3/w (3, 7)	Bulging/extruded/diffuse/protruded discs	GV3, BL23, BL24, BL25, adhesion sites, tender points on the erector spinae/NR/60 × NR × 1.2	NR	NRS, ROM, MRI
		-					
15. Kim et al. (2019)/case report [29]	Ankylosing spondylitis/X-ray	1 (M), 40	3/w (9)	Narrowing disc space	Hard nodules of GB20, GB21, EX-HN1, BL10, BL21, BL22, BL23, BL24, BL25, BL26/NR/80 × NR × 0.75	Lidocaine	NRS, BASFI, ROM, BASDAI, M/K-HAQ
		-					
16. Choi et al. (2021)/case report [19]	HIVD/MRI	1 (F), 57	5/w (39)	Extruded/herniated discs	BL21, BL22, BL23, BL24, BL25, BL26/NR/NR	NR	NRS, ROM, ODI, EQ-5D, physical examination (SLRT, Milgram test), MRI
		-					
17. Cho et al. (2022)/case report [11]	HIVD/MRI	1 (F), 44	5/w (17)	Extruded/herniated discs	BL21, BL22, BL23, BL24, BL25, BL26, etc./NR/80 × NR × 0.75	NR	NRS, ROM, ODI, EQ-5D, physical examination (SLRT, Milgram test), MRI
		-					
18. Wang et al. (2023)/RCT [16]	HIVD/MRI, ultrasonography	Acupotomy + herbal medicine; 30 (M: 14), 48.8 ± 7.48	1/w (3)	Herniated discs	Medial articular process, below base of transverse process, GB30/NR/80 × NR × 1.2, 80 × NR × 1.0	Lidocaine	VAS, JOA, ODI, low back outcome scale
		Herbal medicine; 30 (M: 17), 49.6 ± 5.84					
19. Woo and Cho (2023)/case report [15]	HIVD + FBSS/NR	1 (F), 64	NR (6)	NR	Tender points (vertebral and gluteal regions), lumbar vertebrae/NR/60 × NR × 0.5	NR	NRS, ROM, tenderness response
		-					
20. Sun et al. (2023a)/case report [30]	Acute myelitis/MRI	1 (F), 63	3/w (12)	Abnormal high signal intensity at distal spinal cord, conus medullaris	Lumbar vertebrae, BL31, BL32, gluteus maximus, quadriceps femoris/NR/50 × NR × 0.6	NR	Total sensory score of ASIA
		-					
21. Sun et al. (2023b)/case report [17]	HIVD/MRI	1 (M), 70	1~2/w (20)	Extruded/protruded discs	Internal intervertebral foramen/NR/50 × NR × 0.6	NR	NRS, ODI, ROM, SLRT, MRI
		-					
22. Choi (2023)/case report [31]	Abnormal spinal curvature/X-ray	1 (F), 36	1/w (10)	NR	Tender points/NR/40 × NR × 0.4	NR	VAS, Cobb’s angle (cervical, thoracic, lumbar)
		-					

* Spondylolisthesis, stenosis, herniated lumbar disc, etc. ACU: acupuncture; ASIA: American Spinal Cord Injury Association; BASDAI: Bath Ankylosing Spondylitis Disease Activity Index; BASFI: Bath Ankylosing Spondylitis Functional Index; CCT: controlled clinical trial; CT: computed tomography; d: day; EQ-5D: EuroQol Five-Dimension Questionnaire; FBSS: Failed Back Surgery Syndrome; HIVD: herniated intervertebral discs; JOA: Japanese orthopaedic association low back pain score; LBP: low back pain; M/K-HAQ: Modified/Korean Health Assessment Questionnaire; MRI: magnetic resonance imaging; NDI: Neck Disability Index; no.: number; NR: not reported; ODI: Oswestry Disability Index; RCT: randomized controlled trial; RMDQ: Roland and Morris Disability Questionnaire; ROM: range of movement; SF-36: Short-Form 36-Item Health Survey; SLRT: straight leg raise test; VAS: visual analog scale; w: week.

## Data Availability

No new data were created or analyzed in this study.

## Supplementary Materials

The following supporting information can be downloaded at: https://www.mdpi.com/article/10.3390/healthcare13101141/s1, Table S1: Acupotomy procedures and measures to prevent infection reported in the included studies. Table S2: Safety measures and adverse events reported in the included studies. Refs. [11,12,13,14,15,16,17,18,19,20,21,22,23,24,25,26,27,28,29,30,31,32] are cited in the supplementary materials.
