# Supplementary material for: Diversity and Safety of Acupotomy Treatments for Lumbar Spine Disorders in South Korea: A Review of Clinical Studies
Source: Healthcare (Basel). 2025 May 14;13(10):1141. doi: 10.3390/healthcare13101141 (PMC12110916; doi:10.3390/healthcare13101141)
Supplement: Supplementary file 1 [file healthcare-13-01141-s001.zip › healthcare-3604104-supplementary.pdf]

Supplementary Table S1. Acupotomy procedure and measures to prevent infection reported in the included studies

No. Author (Year)/Study type	Acupotomy procedure and measures to prevent infection
1. Lee et al. (2008)/case series [1]	1. Following rapid penetration through the skin with an acupotomy needle, the incision was made layer by layer until reaching the bone surface. Upon reaching each layer, promptly and resiliently penetrate further after confirming resistance, followed by incision and swift removal.<br>2. Avoiding wide-ranging movements and minimizing stimulation above the bone surface to reduce damage-induced bleeding and mitigates pain.
2. Kwak and Hong (2008) /case series [2]	1. Patient position: supine, lateral decubitus<br>2. The operator wore sterile gloves and disinfected the skin with povidone-iodine solution.<br>3. Align the needle with the direction of muscle, blood vessels, and nerves during insertion and perpendicular to muscles and ligaments during the procedure to minimize damage and maximize therapeutic effects.<br>4. After the procedure, disinfect the area using a povidone-iodine solution, then apply sterile gauze to prevent contamination. Additionally, educate the patient about how to prevent contamination.
3. Jang et al. (2008)/case report [3]	1. Patient position: supine<br>2. Mark the procedure area with a surgical pen, then sterilize it by wiping with iodine tincture and subsequently removing the tincture with 75% alcohol. The operator must wear a disposable mask and surgical latex gloves.<br>3. Insert a marker needle to accurately measure the depth of insertion. Insert the acupotomy needle approximately 2/3 of this depth.<br>4. Use a disposable cupping cup to adequately suction any bleeding from the area for 5 minutes after the procedure. Then, disinfect the area again and apply a patch to prevent contamination.<br>5. On the day following the procedure, dress the area with sterile gauze to assess and maintain the condition of the procedure site. To prevent contamination, avoid washing the area within 3 days and educate on contamination prevention measures."
4. Yun et al. (2010)/RCT [4]	1. Select 5 to 15 sites per session. The acupotomy procedure varied depending on the selected sites.<br>2. Use a probing needle to measure depth, typically insert needles to a depth of 0.5 to 1cm.<br>3. No retention, remove needles after stimulation
5. Jung et al. (2012)/case series [5]	1. Patient position: supine<br>2. Mark the procedure area with a surgical pen, then sterilize the area (using povidone-iodine and alcohol disinfectant) followed by applying a sterile drape. 3. The practitioner should wear surgical gloves, a mask, and a cap.<br>4. Anesthetize was administered using 2ml of 1% lidocaine.<br>5. After the procedure, apply a dressing to prevent secondary infection, ensure the patient rests for about 30 minutes, and then discharge the patient after confirming no adverse findings.
6. Park et al. (2012)/clinical trial [6]	1. Based on the range of positive response points and the patient's reflexes, select 5 to 15 sites per session, with 1 to 3 sessions per patient.<br>2. Sterilize all tools before the procedure: Ensure the treatment table, table cover, washing facilities, autoclave, disinfectant masks, caps, sterile gloves, tweezers, etc., are sterilizable. Nurses are also trained in advance to assist aseptically.<br>3. During the procedure, care must be taken to prevent any complications.<br>4. After the procedure, disinfect with Povidone-Iodine solution and perform manual compression on the area to address any minor bleeding. Attach sterilized gauze and educate the patient on preventing contamination.
7. Sung et al. (2013)/CCT [7]	1. The practitioner wore sterile gloves and a mask, and the procedure area is sterilized with Povidone-Iodine solution.<br>2. Anes Cream (lidocaine + prilocaine; Teaguk, Korea) is applied to the area one hour before the procedure.<br>3. Perform needle insertion up to the periosteum and joint capsule without retaining the needle, employing the single-needle technique. The procedure is conducted with careful monitoring of the patient's pain and sensation to avoid nerve damage, aiming for bleeding after needle removal to relieve congestion in the deep veins caused by the inflammatory response accompanying acute lumbar disc herniation.<br>4. If bleeding occurs post-procedure, apply pressure with sterile gauze until hemostasis is achieved, then disinfect the area again with Povidone-Iodine solution, cover with sterile gauze, and secure with a bandage to complete the procedure.
8. Yuk et al. (2013)/clinical trial [8]	1. Anes Cream was applied to lower back one hour before acupotomy.<br>2. Participants took acetaminophen (Samnam, Korea) to relieve pain during acupotomy.<br>3. The lower back where acupotomy was performed was sterilized in advance.<br>4. After the depth from the skin to the ligament flava had been measured using actual-sized X-ray, the needle was inserted between the spinous process and stimulated the ligament flava. Also, needles was inserted 20 ㎜ - 30 ㎜ apart on both sides of the spinous process and stimulated erect the spinae and the intertransverse ligament which surrounds the facet joint. According to participants, the depth was about 60 - 70 ㎜.<br>5. During the acupotomy, the practitioner checked the participant's pain, and no numbness due to nerve damage was observed.<br>6. The acupotomy site was sterilized, after which sterilized gauze was applied to the site.
9. Kim et al. (2014)/case series [9]	1. Patient position: supine<br>2. The sites were sterilized before the procedure. After the procedure, wet cupping therapy is performed on the site using a disposable sterile cup, followed by sterilization of the site. The area is then secured with gauze

	and a bandage, and the patient is educated on preventing infection at the procedure site.
**10. Kim et al. (2015a)/case series [10]**	1. Patient position: supine<br>2. After the procedure, the procedure site is sterilized and secured with gauze and a bandage.<br>3. The patient is educated on preventing infection at the procedure site.
**11. Kim et al. (2015b)/case series [11]**	1. After the MRI or CT findings had been evaluated, the participant's skin over the corresponding disc level was marked while the patient was in the prone position.<br>2. The participant's lower back was sterilized and anesthetized with lidocaine in advance.<br>3. The acupotomy needles were inserted to depths of 50-60 mm. The practitioner stimulated the soft tissue until the tenderness disappeared. During the treatment, the practitioner checked whether any patients experienced pain and numbness due to nerve damage.
**12. Choi et al. (2017)/case series [12]**	1. Patient position: lying supine with a pillow under the chest area.<br>2. Wearing sterile gloves, reconfirm the stimulation sites and disinfect them using a disposable sterilizing Povidone-Iodine solution.<br>3. The direction of the needle insertion was aligned with the muscle fibers, blood vessels, and nerve pathways, and care was taken not to exceed a depth of 3cm.<br>4. After the procedure, the site was disinfected again with the disposable sterilizing Povidone-Iodine solution and secured with a bandage. The patient was then educated on preventing contamination of the procedure site.
**13. Park et al. (2018)/case report [13]**	After pharmacoacupuncture, acupotomy targeted GV meridian acupoints and L2-L5 articular surfaces, executed at depths under 3.0 cm.
**14. Lee et al. (2019)/case report [14]**	1. Patient position: supine<br>2. Acupotomy treatment was performed on the adhesive area, and the hard area of the soft tissue of the erector spinae muscle, near the acupuncture points.<br>3. The intervention area was disinfected before and after the treatment, and was covered with gauze and bandage following the treatment to prevent infection.
**15. Kim et al. (2019)/case report [15]**	1. Before performing acupotomy, local anesthesia with 0.5 mL of 2% lidocaine hydrochloride salt hydrate was administered on the area surrounding the site for acupotomy. The practitioner disinfected the acupotomy site using a disposable swab soaked in alcohol and a pobidon stick.<br>2. The practitioner wore sterilized latex gloves and a surgical mask before treatment.<br>3. The needles were placed in parallel with the nerves and blood vessels, to a depth of 5 mm -10 mm on hard nodules of the cervical and lumbar vertebrae. Afterwards, a needle was slid in and out, then was taken out.
**16. Choi et al. (2021)/case report [16]**	1. The practitioner wore disposable surgical gloves and mask, and disinfected the treatment area of the patient with alcohol swabs and povidone stick swabs. 2. During the treatment, needle was slid in and out, and then taken out without retention.
**17. Cho et al. (2022)/case report [17]**	1. The practitioner wore disposable surgical gloves and a mask.<br>2. Disinfect the treatment area with alcohol swabs and Povidone stick swabs after the procedure.<br>3. Proceed with needle insertion and remove immediately without retaining the needle.
**18. Wang et al. (2023)/RCT [18]**	1. Patient position: prone<br>2. The table was initially aseptically prepared and draped, and convex array low-frequency probe was wrapped with an aseptic sheath.<br>3. 1% lidocaine was used to perform dermatomic infiltration anesthesia at the located point.<br>5. The acupotomy procedure varied depending on the selected sites.<br>e.g. The insertion was stopped until the tip reached the bone surface of the inferior articular process. The operator slightly raised acupotomy by 1 to 2 mm to make the tip slip from the medial edge of the inferior articular process into the ligament flavum. The operator had a blunt sense of cutting tendinous tissue after sliding through the medial edge of the articular process and controlled the cutting range of 4 to 5mm 4 to 5 times. Ligamentum flavum penetration was denoted by a subtle change of resistance. The release was stopped immediately the numbness radiated to the lower extremities, showing that the tip of the acupotomy had touched the nerve root.
**19. Woo and Cho (2023)/case report [19]**	NR
**20. Sun et al. (2023a)/case report [20]**	1. The acupotomy procedure varied depending on the selected sites.<br>e.g. For intervertebral foramen at L4-5, L5-S1: Position the patient supine with a pillow under the waist to flex the lumbar spine and maximize the space of the intervertebral foramen. Make an incision 0.5 cm lateral from the midpoint between L4-5 and L5-S1, parallel to the sagittal plane, and insert the needle slowly and directly to avoid stimulating the nerve roots. Check for any radiating pain or numbness in the lower extremities while inserting the needle approximately 3-5 cm between the spinous process, the posterior arch of the lumbar spine, and the facet joints. After inserting about 5 cm, where the tissue feels stiff and tough, rotate the needle 90 degrees to perform 5-6 incisions.
**21. Sun et al. (2023b)/case report [21]**	1. patient position: supine, with a pillow under the waist<br>2. Make a parallel incision 0.8 cm lateral and 2 cm below the midpoint between L2 and L5 spinous processes, then directly insert the needle 3 to 5 cm towards the lumbar posterior arch and facet joints. Ensure no radiating pain or numbness occurs to avoid nerve root stimulation. After advancing the needle 5 cm into stiff tissue, rotate it 90 degrees for about 10 incisions.
**22. Choi (2023)/case report [22]**	Acupotomy was performed at the trigger points in the facet areas of L3, L4, and L5.

CCT: controlled clinical trial; NR: not reported; RCT: randomized controlled trial

Supplementary Table S2. Safety measures and adverse events reported in the included studies

No. Author (Year)/Study type	Safety measures	Adverse events
**1. Lee et al. (2008)/case series [1]**	NR	NR
**2. Kwak and Hong (2008) /case series [2]**	NR	NR
**3. Jang et al. (2008)/case report [3]**	NR	Some discomfort and a slight increase in pain at the procedure site were experienced on the day following the procedure, but these symptoms improved within 24 hours.
**4. Yun et al. (2010)/RCT [4]**	NR	NR
**5. Jung et al. (2012)/case series [5]**	NR	NR
**6. Park et al. (2012)/clinical trial [6]**	Infection prevention measures were emphasized, no mention of attention to side effects.	NR
**7. Sung et al. (2013)/CCT [7]**	Compare symptoms and pain before and after the procedure, and document the symptom expression, and check for erythema and warmth to detect possible infection, and perform blood tests if there are signs of infection.	No adverse events
**8. Yuk et al. (2013)/clinical trial [8]**	e.g. There were no instances of sensory numbness due to nerve damage during the procedure.	No sensory numbness
**9. Kim et al. (2014)/case series [9]**	NR	NR
**10. Kim et al. (2015a)/case series [10]**	Observe for any newly emerging symptoms and exacerbation of pain, and monitor the affected area for signs of redness, swelling, and warmth to confirm infection.	No adverse events
**11. Kim et al. (2015b)/case series [11]**	Measurement of Surgical Safety Checklist<br>If any adverse reactions occur during the course of treatment, blood tests will be conducted.	No adverse events
**12. Choi et al. (2017)/case series [12]**	NR	NR
**13. Park et al. (2018)/case report [13]**	NR	Reported localized pain as a side effect, but indicated no interference with daily activities.
**14. Lee et al. (2019)/case report [14]**	NR	NR
**15. Kim et al. (2019)/case report [15]**	NR	NR
**16. Choi et al. (2021)/case report [16]**	NR	NR
**17. Cho et al. (2022)/case report [17]**	NR	NR
**18. Wang et al. (2023)/RCT [18]**	Observe and record any anticipated side effects (local pain, local bleeding, infection, local infection symptoms, nausea, vomiting, dizziness), and provide immediate treatment if necessary.	NR
**19. Woo and Cho (2023)/case report**	NR	NR

**[19]**		
**20. Sun et al. (2023a)/case report [20]**	NR	NR
**21. Sun et al. (2023b)/case report [21]**	NR	NR
**22. Choi (2023)/case report [22]**	NR	NR

References

1. Lee, G.M.; Kim, D.H.; Kim, H.W.; Yeom, S.C.; Kim, H.S.; Kim, D.E.; Yoon, M.J.; Song, D.S.; Yi, K.H. The study on the effect of acupotomy in lumbar HIVD. *Journal of Acupuncture Research* **2008**, *25*, 183-190.
2. Kwak, B.M.; Hong, K.E. Four case of HIVD-lumbar spine patient treated with acupotomy. *J Journal of acupuncture research* **2008**, *25*, 149-156.
3. Jang, E.h.; Kim, S.c.; Lim, N.r.; Na, W.m.; Lim, S.i.; Shin, J.b.; Lee, G.m. Case study of Oriental Medicine treatment with acupotomy tof the herniated lumbar intervertebral disc patient. *The Journal of Korean Acupuncture & Moxibustion Society* **2008**, *25*, 171-181.
4. Yun, J.Y.; Kim, H.W.; Kim, S.S.; Park, S.W.; Kim, E.K.; Lee, G.H.; Lee, G.M. The Clinical Effects of ACupuncture and ACupotomy Therapy for HIVD. *The Journal of Korean Acupuncture & Moxibustion Society* **2010**, *27*, 85-97.
5. Jung, K.Y.; Sur, Y.C.; Jang, W.S.; Lee, J.E.; Kim, K.H.; Shin, G.S.; Han, Y.S. Study and three Cases Report for Lumbar Spinal Stenosis Treatment Using a Combination of Acupotomy and Existing Treatments. *Korean J. Oriental Physiology & Pathology* **2012**, *26*, 120-127.
6. Park, S.W.; Kim, S.S.; Kim, J.Y.; Kim, S.H.; Lee, G.M. The Comparative Study of Effects between Acupotomy and its Cotreatment with Spine Decompression Therapy on HIVD Patients. *The Journal of Korean Acupuncture & Moxibustion Society* **2012**, *29*, 29-39.
7. Sung, I.S.; Yuk, D.I.; Song, D.H.; Kim, M.J.; Hong, K.E. Case Study of with Dochim(刀鍼) & Gwanchim(管鍼) Therapy Combined with Korean Medicine Treatment of Herniated Lumbar Intervertebral Disc Patients. *The Journal of Korean Acupuncture & Moxibustion Society* **2013**, *28*, 153-159.
8. Yuk, D.i.; Sung, I.s.; Song, D.h.; Kim, M.j.; Hong, K.e. Clinical study of lumbar spine stenosis treated by using acupotomy combined with oriental medical treatments. *Journal of Pharmacopuncture* **2013**, *16*, 46.
9. Kim, S.Y.; Kim, H.J.; Ji, Y.S.; Lee, S.M.; Kim, Y.I. The effect of acupotomy on lumbar and cervical spine combined with oriental medical treatment: report of five cases. *J Journal of Acupuncture Research* **2014**, *31*, 183-193.
10. Kim, H.S.; Kim, S.Y.; Kim, H.J.; Kim, E.S.; Kim, Y.I. The effect of acupotomy on lumbar herniated intervertebral disc: report of a case series. *The Acupuncture* **2015**, *32*, 185-195.
11. Kim, H.j.; Jeon, J.h.; Kim, Y.i. Clinical effect of acupotomy combined with Korean medicine: a case series of a herniated intervertebral disc. *Journal of acupuncture and meridian studies* **2016**, *9*, 31-41.
12. Choi, C.W.; Choi, B.S.; Oh, M.S. The Effect of Acupotomy on Traumatic Acute Low back pain : Case Report. *Journal of Haehwa Medicine* **2017**, *26*, 81-87.
13. Park, S.k.; Kim, Y.s.; Jo, H.k.; Yoo, H.r.; Seol, I.c. Case Report: Changes in Magnetic Resonance Imaging in Lumbar Disc Herniation Treated with Korean Medicine. *The Journal of Internal Korean Medicine* **2018**, *39*, 863-869.
14. Lee, Y.J.; Kim, J.I.; Kim, H.B.; Jeon, J.H.; Kim, E.; Kim, Y.I. Cervical and lumbar herniated nucleus pulposus resorption after acupotomy with integrative Korean medicine treatment: A case series of two patients. *Journal of Acupuncture Research* **2019**.
15. Kim, B.S.; Kim, J.I.; Kim, H.B.; Lee, Y.J.; Sung, K.J.; Jeon, J.H.; Kim, E.; Kim, Y.I. A patient with ankylosing spondylitis treated with acupotomy and traditional Korean medicine. **2019**.
16. Choi, H.K.; Lee, Y.R.; Cha, H.J.; Sung, K.J.; Kim, B.S.; Kim, M.J.; Lee, Y.J.; Jeon, J.H.; Kim, Y.I.J.K.J.o.A. Intractable Pain Management by Combined Korean Medicine Treatment Including Acupotomy in Lumbar Disc Herniation: A Case Report. **2021**, *38*, 175-181.
17. Cho, J.I.; Jeon, J.H.; Kim, Y.I. A Case Report of a lumbar herniated intervertebral disc Patient Treated with Korean Medical Treatments, Mainly Managed by Acupotomy. *Journal of Haehwa Medicine* **2022**, *31*, 1-10.

18. Wang, Y.h.; Zhou, Y.; Xie, Y.z.; Fan, X.h.; Liang, W.q.; Wei, X.; Zhao, M.d.; Huo, Y.x.; Zhang, T.; Yin, Y. The effect of ultrasound-guided acupotomy and Juanbi decoction on lumbar disc herniation: A randomized controlled trial. *Medicine* **2023**, *102*, e32622.
19. Woo, J.; Cho, S. A Case Report of Acupotomy Treatment on the Failed Back Surgery Syndrome. *Journal of Korean Medical Society of Acupotomology* **2023**, *7*, 195-201.
20. Sun, T.C.; Kim, H.; Jung, S. A Case Report of Acupotomy Treatment on the Acute Myelitis Patient. *Journal of Korean Medical Society of Acupotomology* **2023**, *7*, 187-194.
21. Sun, T.C.; Choi, J.; Kim, H. A Case Report of Acupotomy Treatment at Internal Intervertebral Foramen on the Chronic HIVD Patient. *Journal of Korean Medical Society of Acupotomology* **2023**, *7*, 15-22.
22. Choi, J. Case Report of Abnormal Spinal Curvature Improved by Acupotomy and Combined Korean Medicine Treatment. *Journal of Korean Medical Society of Soft Tissue* **2022**, *6*, 128-134.
